# The intra-oral variation of salivary ions

**DOI:** 10.1007/s00784-025-06597-7

**Published:** 2025-10-16

**Authors:** Joanita S. van Santen, Zainab Assy, Marja L. Laine, Arjan Vissink, Frans G. M. Kroese, Sarah Pringle, Floris J. Bikker

**Affiliations:** 1 Department of Oral Biochemistry and Periodontology, Academic Centre for Dentistry Amsterdam, University of Amsterdam and Vrije Universiteit Amsterdam, Gustav Mahlerlaan 3004, Amsterdam, 1081 LA The Netherlands; 2 Department of Periodontology, Academic Centre for Dentistry Amsterdam, University of Amsterdam and Vrije Universiteit Amsterdam, Gustav Mahlerlaan 3004, Amsterdam, 1081 LA The Netherlands; 3 Department of Oral and Maxillofacial Surgery, University of Groningen, University Medical Centre Groningen, Hanzeplein 1, Groningen, 9713 GZ The Netherlands; 4 Department of Rheumatology and Clinical Immunology, University of Groningen, University Medical Centre Groningen, Hanzeplein 1, Groningen, 9713 GZ The Netherlands

**Keywords:** Capillary electrophoresis, Electrolytes, Ions, Salivary composition, Whole saliva

## Abstract

**Objectives:**

Dry mouth etiologies, which often present as a combination of hyposalivation and xerostomia, are difficult to differentiate. Employing a combination of a location-based approach in the oral cavity and salivary ion measurement may resolve this differential diagnostic gap. In this light, the aim of this cross-sectional study was to determine the concentrations of salivary sodium, potassium, chloride, phosphate, calcium, ammonium, nitrate, nitrite, fluoride, sulphate and magnesium at 7 intra-oral locations in healthy subjects (*N* = 30).

**Methods:**

First, sterile foam tipped applicators were selected as the most suitable tool to take saliva samples from these areas. Ion levels were analyzed using a capillary electrophoresis system.

**Results:**

Almost every ion included showed a unique intra-oral distribution pattern across the selected regions. For example, the palate demonstrated the highest sodium concentration and in contrast, the floor of the mouth showed the lowest. The observed distribution patterns were consistent between individuals.

**Conclusions:**

Local variations in salivary ion concentrations may be influenced by the positioning of the salivary glands and their associated ducts but could also relate to their function in the oral cavity.

**Clinical Relevance:**

The results from our healthy controls can form a reference for future studies and potentially for diagnostic purposes in clinical contexts.

**Supplementary Information:**

The online version contains supplementary material available at 10.1007/s00784-025-06597-7.

## Introduction

Dry mouth relates to both xerostomia, the subjective feeling of a dry mouth, and hyposalivation, the objective decrease of saliva production. It is important to distinguish the two concepts from each other, considering that an individual can experience the feeling of mouth dryness, but not necessarily display a reduction in saliva production [1, 2]. Differences in the subjective experience and objective measurement of a dry mouth are linked to the various etiologies of oral dryness. These etiologies include causes such as radiotherapy of the head and neck region, polypharmacy, Sjögren’s disease (SjD), dehydration, aging, stress and anxiety [3–6].

The Xerostomia Inventory (XI) is commonly used to quantify the severity of xerostomia [7]. The XI is an 11-item questionnaire with which patients rate their overall experience with xerostomia symptoms on a 5-point Likert scale. In general terms, the easiest way to objectively analyze hyposalivation is by measuring the amount of saliva produced within a certain time frame, also known as the flow rate (FR) [8]. However, neither of these methods distinguish between the different causes of a dry mouth. Therefore, Assy et al. expanded the available tools for assessing dry mouth by introducing the Regional Oral Dryness Inventory (RODI), a method focused on evaluating perceived dryness in different areas of the mouth [9]. The RODI results in a score for how patients experience dry-mouth symptoms at each of the 9 different locations in the mouth: the upper and lower lip, the anterior and posterior palate, the anterior and posterior tongue, the floor of the mouth, inside of the cheeks, and the pharynx [9]. Research using the RODI demonstrated differences in dry-mouth perception at various intra-oral locations [10], for example, SjD patients experienced the posterior part of the palate as significantly drier compared to the other patient groups and the healthy controls [10]. Despite being able to differentiate between some dry-mouth etiologies, the observed differences were insufficient to be able to specifically diagnose or screen for these conditions. Additionally, it should be noted that the RODI reflects subjective parameters rather than objective ones.

Therefore, interest has also turned to salivary biomarkers to aid in the objective analysis of various dry-mouth conditions. In this light, specific attention has been paid to salivary ions and the variation in their concentration, measured in whole or glandular-specific saliva, when comparing healthy individuals to other dry-mouth patients [11–16]. However, these differences in concentration are not specific enough to each patient group to be used for diagnostic purposes [16]. We hypothesize that combining the two approaches measuring the concentrations of salivary ions in saliva collected from different regions of the mouth may further increase the level of sialometric resolution and ultimately enable differentiation of patients with various etiologies of dry mouth from each other. The current study aimed to create a reference of the ion composition at the various intra-oral locations in healthy individuals, to serve as a basis for future research in salivary ions for screening and/or diagnostic purposes.

## Methods

### Selection saliva collection tool

A variety of tools were compared for suitability to sample and analyze saliva from various intra-oral surfaces. Tools assessed included Sialopapers (Oraflow Inc., New York, USA), Bio Schirmer strips (Opto Medica Oftalmologia Srl, Rome, Italy), ISOHELIX™ DNA/RNA Buccal Swabs - SK-2S (Isohelix, Kent, UK), Sterile Foam Tipped Applicators (Puritan, Guilford, Maine, USA), Sugi Sponge Points (Questalpha GmbH & Co. KG, Eschenburg, Germany) and Invitrogen iBlot2 Absorbent Pad mini (cut into pieces of 1 cm^2^) (Thermo Fisher Scientific Inc., Massachusetts, USA). These tools were evaluated for their absorption and retention properties, and presence of inherently contained ions. First, the maximum absorption of the tools was determined by submerging them in unstimulated saliva collected as described below (Sect. 2.3), for 5 min and measuring the maximum amount of saliva absorbed by weight. Next, the retention volume was determined by adding 200 µL of saliva to an unused tool, followed by centrifugation at 20,018×*g*, for 5 min. at room temperature (RT) and measuring the amount of saliva eluted, by weight. The volume of saliva still in or on the tool, measured by weight, was considered the retained saliva. Lastly, inherent ion contamination was determined by soaking unused tools in 500 µL of MiliQ water for 10 min while on a shaker (Heidolph Titramax 100; Heidolph Instruments GmbH & Co. KG, Germany) at speed setting 7. Afterwards the ion content of the eluent was analyzed undiluted by capillary electrophoresis following the method described in Sect. 2.4. The final tool selected for use, was again tested for ion contamination to assess if any differences could be observed between different batches (10 tools from 2 batches).

### Study population

The study was approved by the local Ethical Examination Committee at the Academic Centre for Dentistry Amsterdam (ACTA) (protocol number: OBC-2023). Healthy participants were recruited amongst the employees of the ACTA, between October and December 2023. Prior to participation, all volunteers received an information letter and signed an informed consent form. Inclusion criteria were adults over the age of 18 years old. Exclusion criteria were systemic diseases and the use of any medication, besides anticonception drugs, prescribed topical medication, and prescribed eye drops. No information about the participants oral hygiene practices was requested, nor were the participants asked to standardize the oral hygiene practices prior to participation in the study.

### Saliva collection

Saliva samples were collected between 9:00 and 11:00 h. Participants were instructed to refrain from eating, drinking and brushing their teeth for one hour prior to the collection procedure. Before collection, all participants were asked to rinse their mouth with 30 mL of water which was spat out and followed by a 10 min break. In addition, after swallowing one last time, unstimulated whole saliva (UWS) was collected, by instructing the participants to sit straight and relaxed, and letting their saliva pool in their mouth for 5 min. The pooled saliva was drooled into a medicine polypropylene-cup (Greiner Bio-One International GmbH, Alphen aan den Rijn, Netherlands). If participants could not hold the saliva in their mouth during the 5 min, they were allowed to expectorate the saliva in between as well. This was followed by a 5 min break, whereafter participants were asked to swallow, followed by the collection of saliva using the sterile foam tipped applicators, from the following intra-oral locations: hard and soft palate; anterior tongue; posterior tongue, upper cheek; middle cheek; lower cheek; and the floor of the mouth. The locations were swabbed in the order and manner as indicated in Figure S1. Collection per region was performed for 30 s with a 30 s break in between each region. Participants were also asked to swallow once immediately before each region was sampled.

Before and after collection the cups were weighed to determine the salivary FR (in mL/min) of the UWS. Next, the swabs were weighed before and after collection, to determine the absorption (in µL) of saliva by the swabs per region. To determine the saliva volume, it was assumed that 1 mg of saliva is equal to a volume of 1 µL. This was followed by centrifugation at 20,018 ×*g* for 5 min at RT. The supernatant was then collected and aliquoted into Eppendorf tubes and stored at −20 ℃.

### Capillary electrophoresis

To determine the ion concentrations, all samples were thawed and centrifuged at 20,018 ×*g* for 5 min at RT, to remove any potential debris. The supernatant of the UWS samples was diluted 1:10 and the supernatant of the regional samples was diluted 1:100, both in MiliQ water. Cations and anion in all saliva samples were measured by a capillary electrophoresis (CE) system (CAPEL-205, Lumex Instruments, Canada) equipped with a cassette containing a 60 cm capillary with an inner diameter of 75 μm (BGB Analytik Benelux B.V., Harderwijk, the Netherlands). For cation and anion measurements, the manufacturers protocols for the “cations in water with capel” test kit (Lumex Instruments, Canada) and the “anions in water with capel” (Lumex Instruments, Canada) test kit were followed, respectively. The analysis time for the cation analysis was adjusted to 7 min. The resulting electropherograms were interpreted making use of the elforun-205 software (Envico, Zoeterwoude, the Netherlands). Ion concentrations were denoted in mM.

### Data analysis

To determine if there were any differences between the medians of the ion concentrations in the various saliva samples, a Friedman test was performed, followed by the Wilcoxon signed-ranks test with a Bonferroni correction (α = 0.00179), to determine which locations differed significantly from each other. Both tests were conducted making use of R [17] in Rstudio [18] with the “rstatix” package [19]. To investigate the effect of sex at the specific intra-oral locations on the measured ion concentrations in UWS and at the locations, multivariate linear regression (α = 0.05) was conducted with the IBM SPSS software [20]. Figures were created making use of Graphpad Prism version 8.1.0 for Windows (Boston, Massachusetts USA). Any capillary electrophoresis data below the detection limit, were substituted by an arbitrary value of 0.00 mM. For outcome measurements with more than 5 substituted values, no multivariate linear regression was performed.

## Results

### Selection saliva collection tool

Six different tools were tested for their potential effectiveness as a saliva collection tool for specific locations in the mouth. These tools were selected based on their known application in saliva diagnostics, their common use in a laboratory setting and/or their absorbent properties [21–23].

For the analysis of the saliva from the various regions in the mouth, it was of importance to obtain a minimum volume of 5 µL for capillary electrophoresis and to ensure that the retention of the saliva in the collection tool after centrifugation was minimal. Therefore, the tools were tested for their maximum absorptive volume and the saliva retention in the tool after centrifugation (Table 1). The tool with the highest absorption was the Sugi Sponge Points with an average maximum absorption of 493.34 µL saliva. The tool with the lowest retention was the Sialopaper, with an average retention of 4.84 µL of saliva.

**Table 1:** Characteristics of the various saliva collection tools

Tools	Mean (SD) max. absorption (µL) (*N* = 4)	Mean (SD) retention (µL) (*N* = 3)	Mean (SD) ion contamination (mM) (*N* = 3)
Sialopaper	44.85 (16.89)	4.84 (0.16)	Na^+^=2.19; Ca^2+^=0.83; Cl^−^=1.05; SO_4_^2−^=1.97; NO_3_^−^=1.46
ISOHELIX™ DNA/RNA Buccal Swabs	72.47 (22.44)	12.75 (6.03)	K^+^=3.58; Na^+^=12.96; Cl^−^=6.41; SO_4_^2−^=35.85; NO_3_^−^=4.82; PO_4_^3−^=0.06
Sterile Foam Tipped Applicators	112.66 (17.39)	7.46 (0.75)	PO_4_^3−^=0.03 (*N* = 13)
Bio Schirmer Strips	204.15 (76.22)	22.62 (8.73)	NH_4_^+^=2.67; Na^+^=0.63; Ca^2+^=1.52; Cl^−^=2.52; NO_3_^−^=6.99; F^−^=0.29; PO_4_^3−^=0.10
Invitrogen iBlot2 Absorbent Pad mini	472.80 (72.22)	108.11 (7.65)	K^+^=9.25; Na^+^=0.83; Cl^−^=2.10; PO_4_^3−^=0.15
Sugi Sponge Tips	493.43 (54.36)	91.95 (1.92)	Na^+^=7.52; Ba^2+^=1.58; Cl^−^=4.27; SO_4_^2−^=3.43;

*Max. = maximal*

In addition, the tools were tested for the initial presence of ions i.e., contamination (Table 1). The sterile foam tipped applicators displayed the least amount of ion contamination, compared to the other tools (0.03 mM). In light of the average phosphate concentration in the UWS or at any of the locations was 13.42 mM, we considered the contamination on the sterile foam tipped applicators negligible. Considering the ease of handling, their mid-range absorption, low retention volume and minimal contamination in particular, the sterile foam tipped applicators were selected as the best saliva sampling tool for the various intra-oral locations.

### Participant characteristics and intra-oral saliva collection

A total of 30 participants were included (Table 2). The average age of the participants was 38 years (range = 24–64 years) and 67% of all participants were female. Amongst the included participants, one had irritable bowel syndrome and one participant rhinitis, eczema and glaucoma. The median UWS FR was 0.29 mL/min (IQR = 0.21–0.45 mL/min).

**Table 2:** Characteristics of the participants (UWS: unstimulated whole saliva)

	Participants (*n* = 30)
Sex (Male: Female)	12:18
Age (mean (range)) (years)	38 (24–64)
UWS flow rate (median (IQR)) (mL/min)	0.29 (0.21–0.45)
Absorption per location: (median (IQR)) (µL)	
Palate	15.5 (10.0–22.7.0.7)
Anterior tongue	22.0 (16.5–44.7)
Posterior tongue	36.0 (28.2–52.0)
Upper part of the cheek	38.0 (27.2–56.0)
Middle part of the cheek	19.3 (13.3–26.6)
Lower part of the cheek	35.5 (27.2–53.6)
Floor of the mouth	78.8 (48.8–109.9.8.9)

Regarding the saliva collection with the sterile foam tipped applicators, the results showed that the lowest volume of saliva was obtained from the palate, with a median absorption of 15.5 µL (IQR = 10.0–22.7.0.7 µL; Table 2). The highest volume of saliva was obtained from the floor of the mouth with a median absorption of 78.8 µL (IQR = 48.8–109.9.8.9 µL).

### Ion concentrations at the various intra-oral locations

**Fig. 1:**
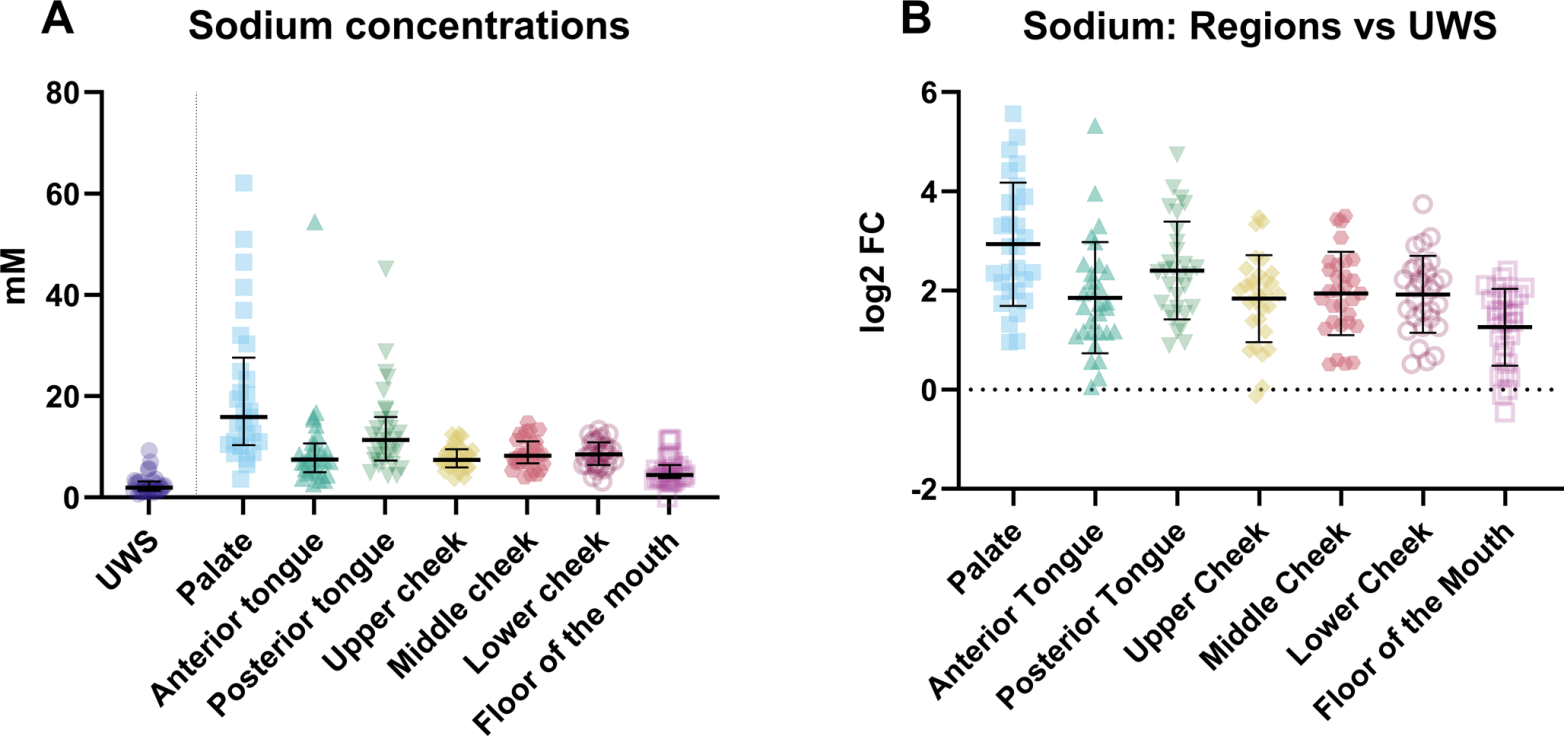
(**A**) The sodium concentrations in unstimulated whole saliva (UWS) and at the various locations in the mouth and (**B**) the difference in sodium concentration between the various regions and unstimulated whole saliva (UWS) expressed as the log^2^ fold change (log2FC)

For each cation, there was a different pattern of distribution in the oral cavity. For sodium, the highest concentrations among all sampled locations were found on the palate (median = 15.86 mM, IQR = 10.34–27.61 mM) and on the posterior part of the tongue (median = 11.32 mM, IQR = 7.25–15.90 mM), making these locations significantly different from the others (Fig. 1; Table S1). The lowest concentration of sodium at the locations was found on the floor of the mouth (median = 4.44 mM, IQR = 3.81–6.40 mM; Fig. 1), also significantly different from the concentrations at the other specific intra-oral locations (Table S1).

**Fig. 2:**
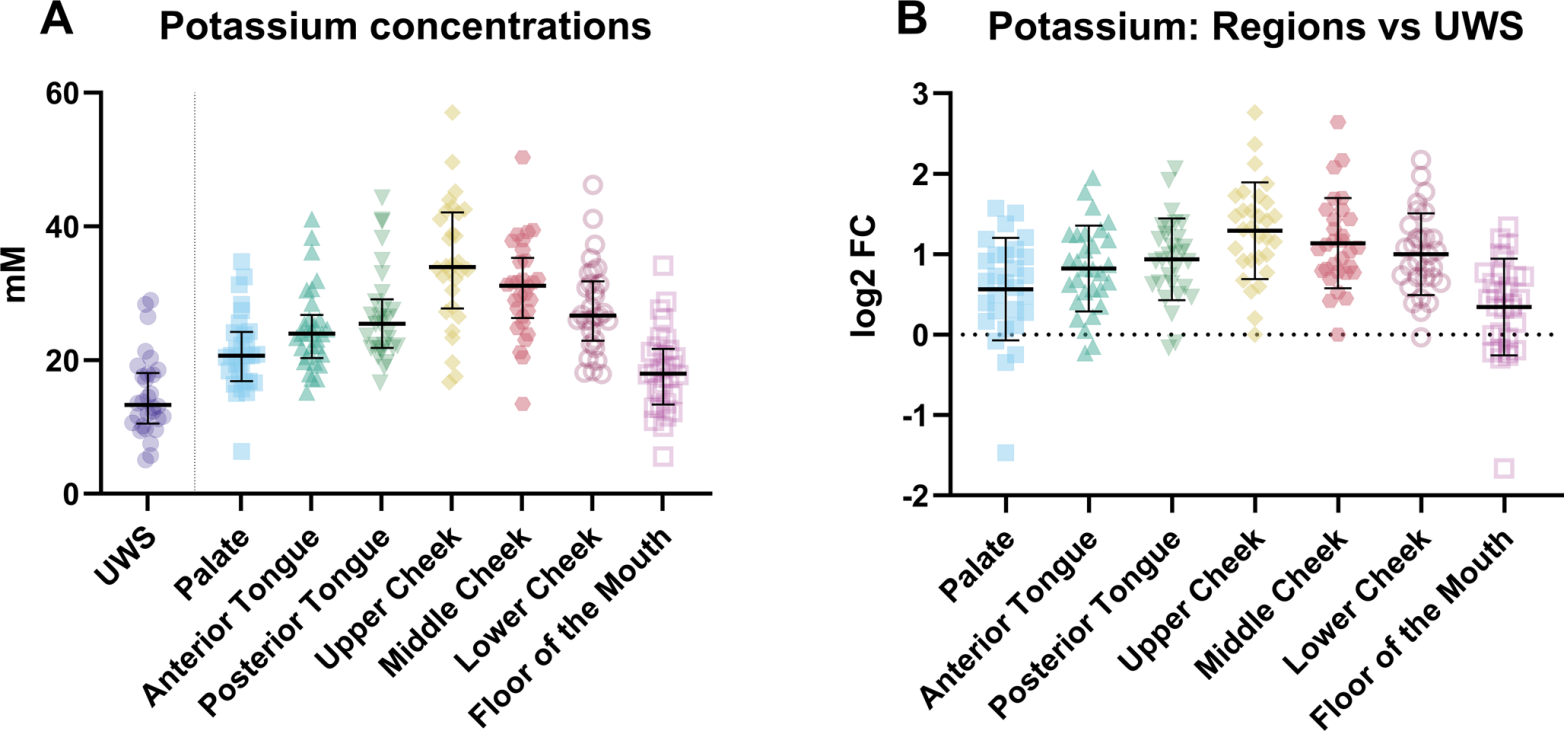
(**A**) The potassium concentrations in unstimulated whole saliva (UWS) and at the various locations in the mouth and (**B**) the difference in potassium concentration between the various regions and unstimulated whole saliva (UWS) expressed as the log^2^ fold change (log2FC)

Potassium depicts a different pattern as opposed to sodium, with the highest concentration on the upper cheek (median = 33.89 mM, IQR = 27.73–42.08 mM) and the potassium concentration being significantly lower on the palate (median = 20.64 mM, IQR = 16.83–24.19 mM) and the floor of the mouth (median = 17.98 mM, IQR 13.34–21.65 mM) compared to the other regions (Fig. 2; Table S2). All regions differed significantly from UWS (Fig. 2B).

**Fig. 3:**
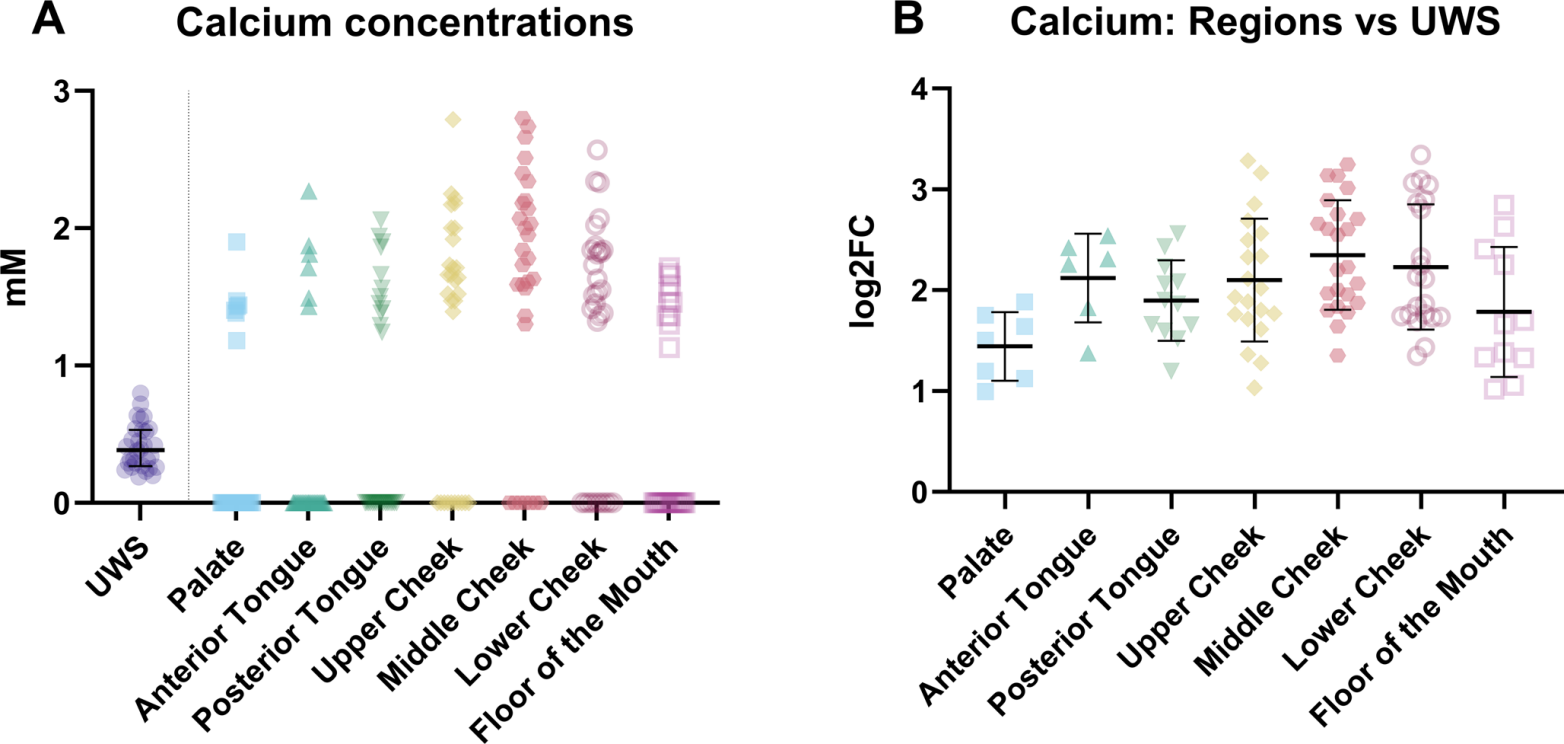
(**A**) The calcium concentrations in unstimulated whole saliva (UWS) and at the various locations in the mouth and (**B**) the difference in calcium concentration between the various regions and unstimulated whole saliva (UWS) expressed as the log^2^ fold change (log2FC)

For multiple samples from the various intra-oral locations, the calcium concentrations were below the detection limit of the capillary electrophoresis system. The number of data points below the detection limit (N) for each location were as follows: *N* = 22 for the palate; *N* = 24 for the anterior tongue; *N* = 18 for the posterior tongue; *N* = 10 for the upper part of the cheek; *N* = 7 for the middle part of the cheek; *N* = 9 for the lower part of the cheek; *N* = 19 for the floor of the mouth. This noted, enough data could still be obtained for the upper, middle, and lower part of the cheek. These are probably the locations with the highest concentrations (Fig. 3). Furthermore, it seems likely that the calcium concentrations at the intra-oral locations are higher than in UWS (Fig. 3B; Table S3).

**Fig. 4:**
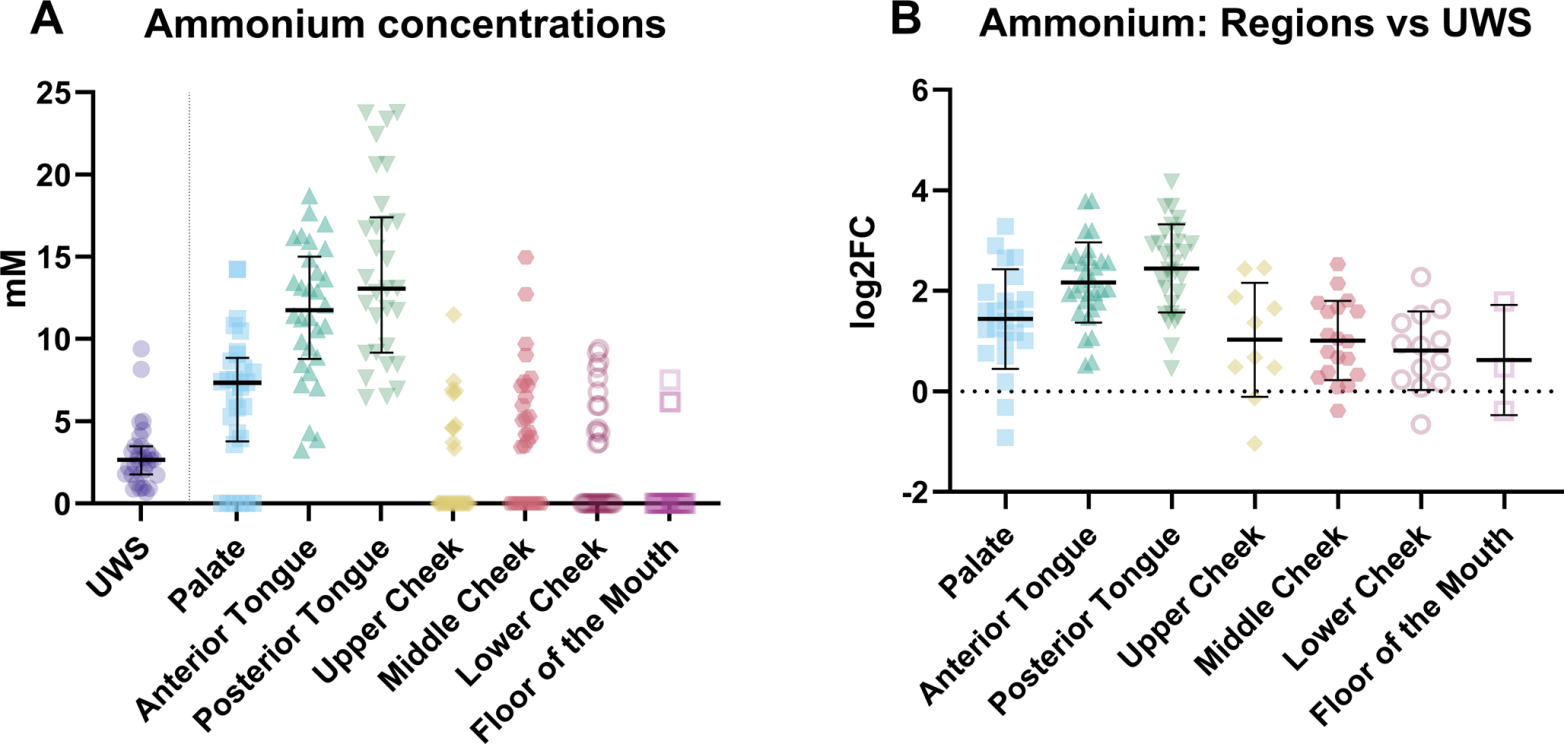
(**A**) The ammonium concentrations in unstimulated whole saliva (UWS) and at the various locations in the mouth and (**B**) the difference in ammonium concentration between the various regions and unstimulated whole saliva (UWS) expressed as the log^2^ fold change (log2FC)

The highest ammonium concentrations can be found on the anterior (median = 11.73 mM, IQR 8.77–15.01 mM) and posterior tongue (median = 13.06 mM, IQR = 9.17–17.39 mM; Fig. 4). While lower ammonium concentrations were detected at the three cheek areas and the floor of the mouth (Fig. 4; Table S4). It is important to note, as for calcium, that there are some missing data points for the palate (*N* = 6), the upper part of the cheek (*N* = 20), middle part of the cheek (*N* = 11), lower part of the cheek (*N* = 17) and on the floor of the mouth (*N* = 27) for the ammonium concentration. Therefore, regarding the distribution of ammonium at these locations in the mouth, no concrete conclusions can be drawn.

**Fig. 5:**
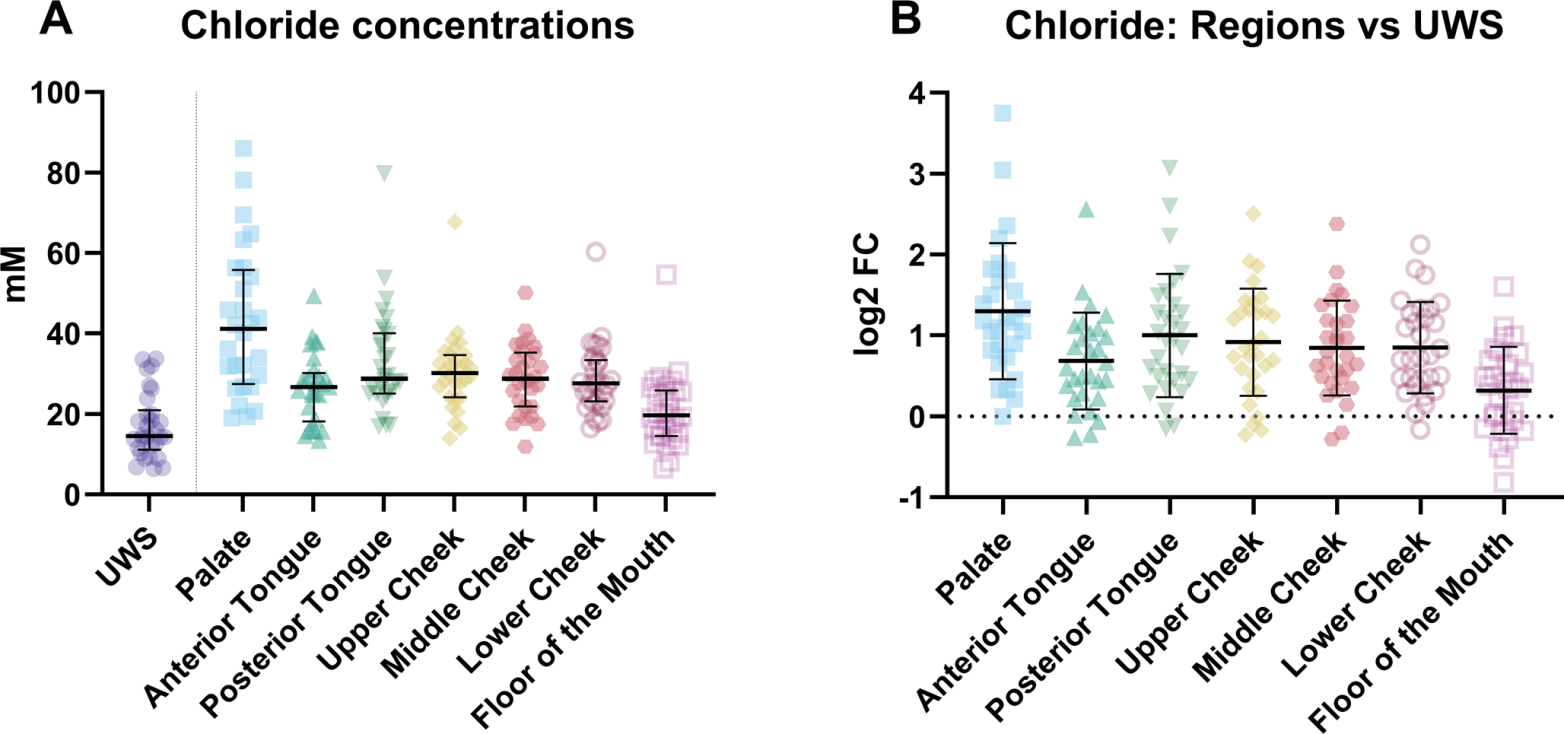
(**A**) The chloride concentrations in unstimulated whole saliva (UWS) and at the various locations in the mouth and (**B**) the difference in chloride concentration between the various regions and unstimulated whole saliva (UWS) expressed as the log^2^ fold change (log2FC)

Analysis of anion concentrations, beginning with chloride, showed that the highest concentration was found on the palate (median = 41.08 mM, IQR = 27.49–55.75 mM) and the lowest on the floor of the mouth for the locations (median = 19.71 mM, IQR = 14.54–25.89 mM; Fig. 5). For chloride there was no significant difference between the concentration on the floor of the mouth and in the UWS (Table S5, Fig. 5B). The pattern of chloride ion content seems to resemble that of sodium and shows that chloride concentrations are comparable to the combined concentrations of sodium and potassium.

**Fig. 6:**
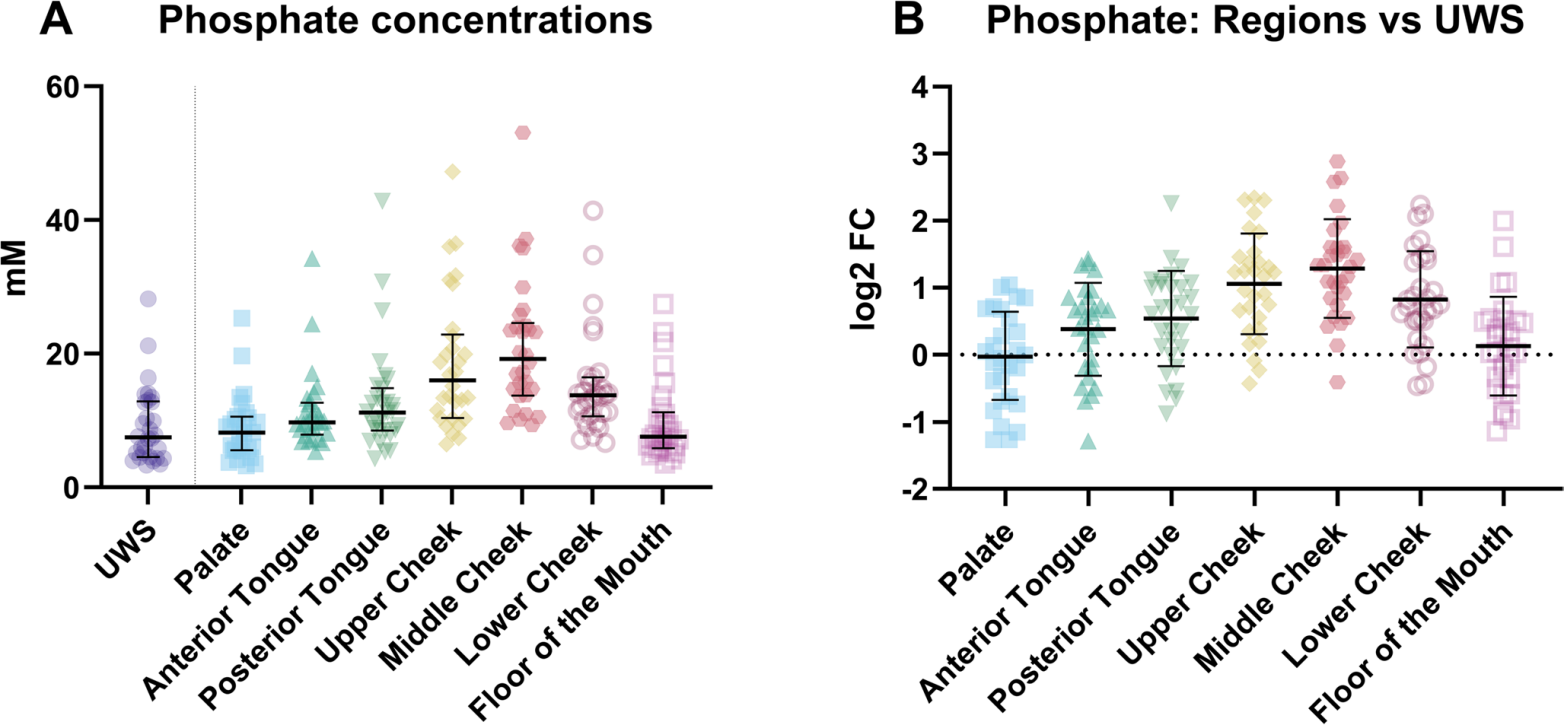
(**A**) The phosphate concentrations in unstimulated whole saliva (UWS) and at the various locations in the mouth and (**B**) the difference in phosphate concentration between the various regions and unstimulated whole saliva (UWS) expressed as the log^2^ fold change (log2FC)

For phosphate, the distribution was different than chloride. The highest concentration was found for the upper and middle part of the cheek, respectively median = 16.02 mM (IQR = 10.37–22.83 mM) and median = 19.20 mM (IQR = 13.75–24.58 mM) (Fig. 6; Table S6). The lowest phosphate concentration was found on the floor of mouth and the palate (Table S6). The phosphate concentration at these locations differed significantly from UWS only for posterior tongue, upper, middle, and lower cheek (Fig. 6B).

**Fig. 7:**
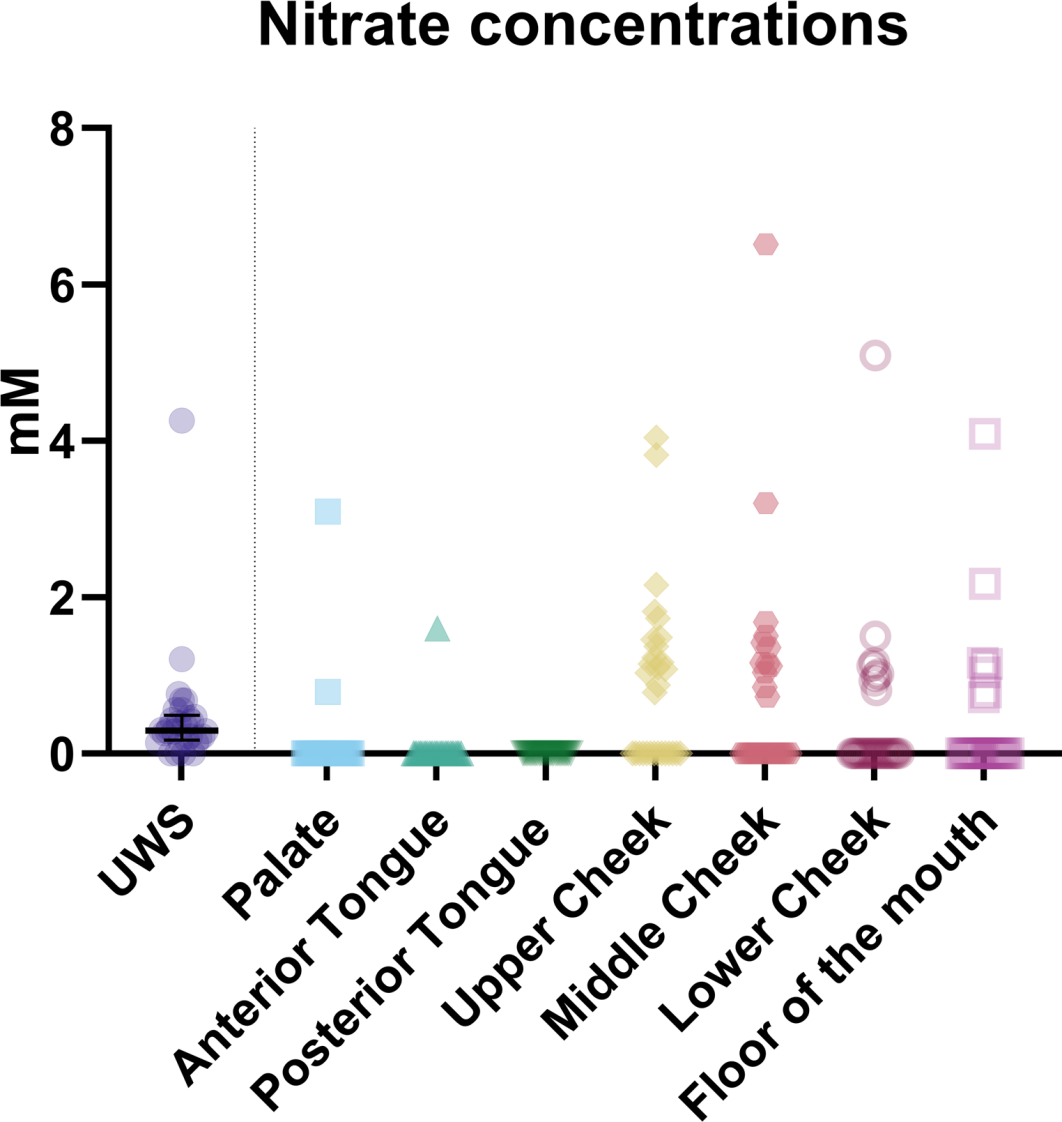
The nitrate concentrations in unstimulated whole saliva (UWS) and at the various locations in the mouth

Nitrate is one of the ions with the lowest concentrations in saliva, which resulted in a relatively high number of undetectable datapoints. The locations with the most points below the detection limit were the palate and the anterior and posterior parts of the tongue (Fig. 7; Table S7). The number of data points below the detection limit (N) for each location were as follows: *N* = 28 for the palate; *N* = 29 for the anterior tongue; *N* = 30 for the posterior tongue; *N* = 13 for the upper part of the cheek; *N* = 18 for the middle part of the cheek; *N* = 21 for the lower part of the cheek; *N* = 23 for the floor of the mouth.

Nitrite, fluoride, sulphate and magnesium were sometimes detected in the UWS of the participants, but at low concentrations limiting reliable analysis (Table S8). An overview of all the median ion concentrations per regions can be found in Figure S2.

Lastly, multivariate linear regression was performed to determine if there were any sex-linked differences between the ions at various locations. Only for potassium an association was found between being female and having a lower potassium concentration on the floor of the mouth (Unstandardized B = −4.469, *p* = 0.026).

## Discussion

The current study aimed to map concentrations of salivary ions at 7 intra-oral locations in healthy individuals, serving as a foundation for future research on regional salivary ions for screening and/or diagnostic purposes. It was found that each ion has a distinct concentration pattern at various intra-oral locations (Table S9).

Out of the selected tools, the sterile foam-tipped applicators appeared to be the most appropriate sampling tools for collecting saliva from various intra-oral locations. It has to be noted that the foam-tipped applicators contained an inherent phosphate contamination of 0.03 mM (Table 1). Yet as the average phosphate concentration at the various regions was 13.42 mM, we considered this negligible. Capillary electrophoresis was selected for its ability to analyze small sample volumes. Although flame photometry and atomic absorption spectroscopy are often the techniques of choice to analyze the salivary cations, they seem less suitable for analyzing anions [16, 24]. In this respect, and considering its high sensitivity, ability to analyze low sample volumes and wide range of ions which it can detect (both cations and anions), capillary electrophoresis was deemed more suitable [25–27].

The ranges of ion concentrations for sodium, chloride, potassium, ammonium, and phosphate across different regions showed consistent patterns among individuals (Figure S3). It seems plausible that their local levels are partly due to the secretion rates and levels of their gland of origin as well as by the proximity to the orifices these respective glands. A pertinent example of this would be potassium; demonstrating the highest concentration on the upper cheek, a location close to the parotid duct exit. The potassium concentration at this location (Fig. 2) appears to be close to the known concentration of potassium in parotid saliva (± 46 mM) [28]. Other examples are phosphate and calcium displaying the highest concentration at the cheek regions.

It could also be speculated that the differences in ion distribution could relate to their function. Sodium levels are highest at the palate and posterior tongue, where it may help regulate taste sensitivity and fluid balance. This process likely involves epithelial sodium channels in the tongue and palatal taste buds, with sodium concentrations potentially serving as an activation threshold for these receptors [29–32]. Interestingly, relatively high ammonium levels were found on the tongue. Ammonium, which is a cation, is not secreted by the salivary glands. Instead its origin can be traced back to ammonia-producing bacteria, which mainly use arginine and urea as substrates for ammonia production [33]. Despite the production of ammonia by the bacteria, ammonia is converted to ammonium at a lower pH (< pH 8.0). The pH of UWS, namely between 6.4 and 6.9, likely facilitates this conversion to ammonium [34]. Known ammonia producing oral bacteria include a variety of *Streptococci*, some *Lactobacilli* and others, which can be mainly found in dental plaque and tongue biofilm [35–38]. The reverse applies to nitrate, which likely has the lowest concentrations on the palate and the anterior and posterior parts of the tongue. This may be attributed to the presence of nitrate reducing bacteria, primarily on those surfaces [39–41]. The nitrate detected in saliva has its origin from food sources, mainly fruits and vegetables, and is found at concentrations 10-fold higher in saliva than in blood plasma [42, 43]. This may provide an explanation for increased nitrate presence on the cheeks, considering that the parotid exit is in this location.

In this study, we mapped the ion distribution at various intra-oral locations in healthy volunteers. Possibly, this pattern of ion distribution differs in patients with dry-mouth conditions. Dry-mouth patients exhibit a reduced salivary flow rate, lower pH, diminished buffering capacity, and impaired viscoelastic, hydrating, and lubricating properties due to altered mucin glycosylation [44]. Furthermore, a previous systematic review with meta-analysis demonstrated that dry-mouth patients, particularly those with Sjögren’s disease, exhibited significantly higher concentrations of sodium, chloride, and calcium ions in UWS compared to healthy controls [16]. These higher ion levels in UWS could also affect the ion levels at the various intra-oral locations or could cause a shift in ion levels at specific locations. Therefore, further research is required to elucidate ion distribution in the intra-oral regions studied here, in dry-mouth patients.

A limitation of this study is that in some instances only a low volume of saliva could be obtained, especially from the palate. This is also the main reason why the palate was not subdivided into the hard palate and the soft palate, despite their anatomical differences [45]. Additionally, although the capillary electrophoresis system did allow for measurements with low sample volumes (5 µL), it has proven to not be ideal for some of the ion analyses (nitrate, nitrite, magnesium and sulphate). Abundant ions, (sodium, chloride, and potassium) could be reliably measured without any issue. If the focus of future research is on any of the less abundant ions, it might be necessary to utilize a different analysis technique such as ion exchange chromatography with a high-performance liquid chromatography system. Furthermore, in the present study the RODI was not employed [9, 10]. It would be of interest to include the RODI alongside the intra-oral salivary ion measurements for future studies investigating dry-mouth patients, to determine whether there are any associations and/or correlations, between the experience of dryness and the ion measurements at the locations. We also did not assess the oral health status of the participants. For future research it could be of interest to include parameters such as the gingival index, plaque index, number of natural teeth and/or implants etc. Lastly, it could be of value to assess whether these results hold up over time within individuals, by performing a longitudinal assessment. However, we assume that will not impact the results, considering that the observed ion distribution patterns are consistent between individuals.

## Conclusion

In summary, the present study demonstrates the development of an intra-oral saliva collection method for the analysis of location-based salivary ion concentrations. Making use of the Sterile Foam Tipped Applicators, 7 intra-oral locations were successfully sampled. Furthermore, capillary electrophoresis has proven to be a reliable technique to be utilized for salivary ion measurements in UWS and for the more abundant salivary ions in low volume saliva samples of healthy individuals. The results have shown that each ion has a distinct intra-oral concentration pattern which is similar across individuals. Local variations may be influenced by the positioning of the salivary glands and their associated ducts and anatomical structure differences in the oral cavity, but could also be related to their roles in oral health, which requires further investigation. All in all, the observed ion patterns can potentially be used as healthy reference levels for future studies looking into the ion distribution in specific patient groups, such as Sjögren’s disease patients, for diagnostic purposes.

## Supplementary Information

Below is the link to the electronic supplementary material.

Supplementary Material 1 (DOCX 830 KB)

## Data Availability

The processed ion concentration data has been published and can be accessed through the following DOI: https://doi.org/10.48338/VU01-NWN11E. The data is available upon request for reuse.
