# Supplementary material for: The intra-oral variation of salivary ions
Source: Clin Oral Investig. 2025 Oct 16;29(11):515. doi: 10.1007/s00784-025-06597-7 (PMC12528311; doi:10.1007/s00784-025-06597-7)
Supplement: Supplementary file 1 — Supplementary Material 1 (DOCX 830 KB) [file 784_2025_6597_MOESM1_ESM.docx]

**Supplementary**

**
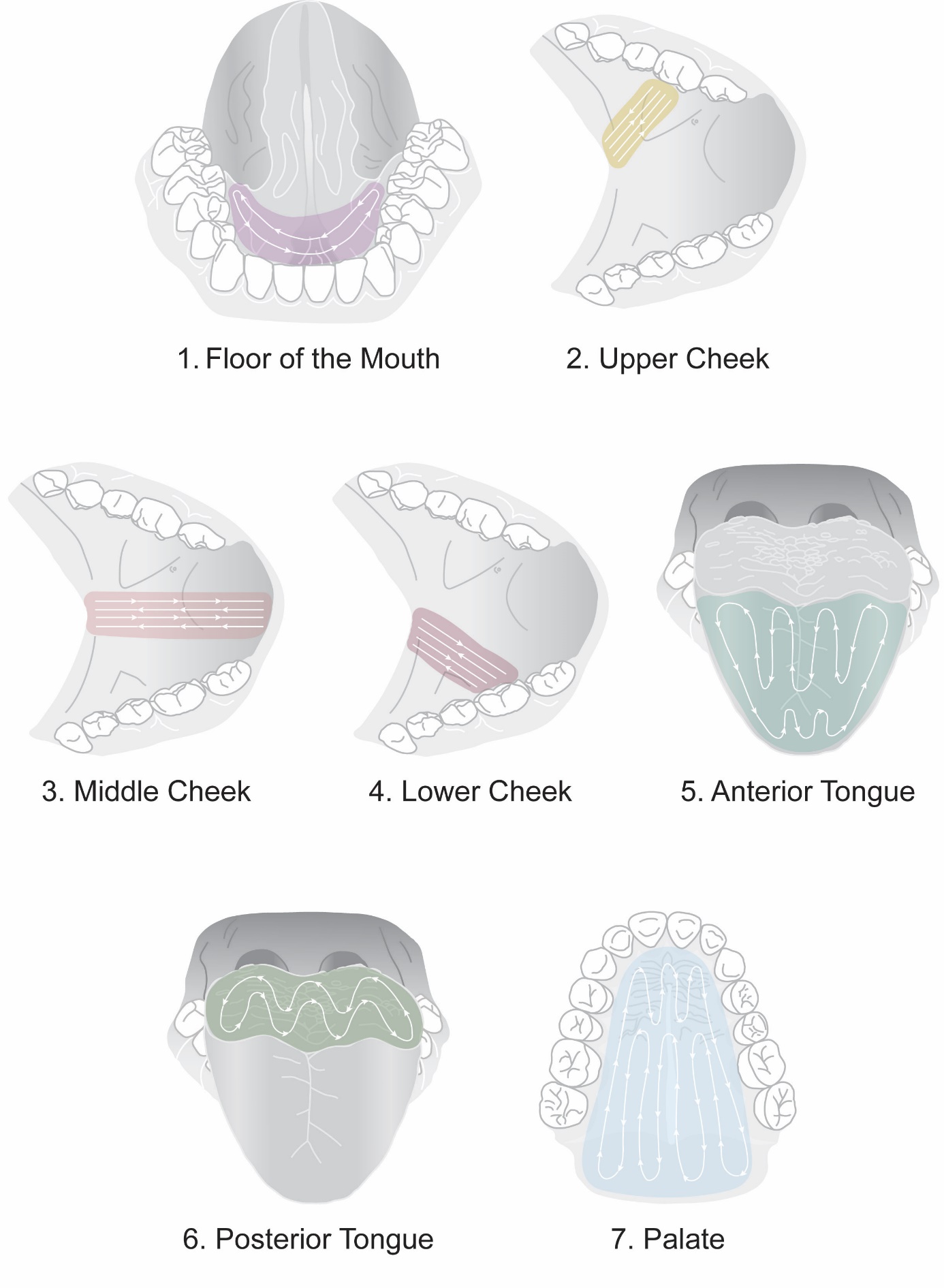
**

*Figure S1. Illustrations indicating the manner of swabbing each intra-oral location in order (1-7).*

*Table S1. Significant differences in the sodium concentration between the various sources of saliva by paired Wilcoxon signed rank test with Bonferroni correction.*

| **Sodium** | Palate | Anterior tongue | Posterior tongue | Upper cheek | Middle cheek | Lower cheek | Floor of the mouth |
| --- | --- | --- | --- | --- | --- | --- | --- |
| UWS | **** | **** | **** | **** | **** | **** | **** |
| Palate | - | **** | * | **** | **** | *** | **** |
| Anterior tongue | - | - | *** | NS | NS | NS | ** |
| Posterior tongue | - | - | - | * | ** | * | **** |
| Upper cheek | - | - | - | - | NS | NS | *** |
| Middle cheek | - | - | - | - | - | NS | **** |
| Lower cheek | - | - | - | - | - | - | **** |

NS = Not Significant

UWS = Unstimulated Whole Saliva

* = p < 1.79*10^-3^

** = p < 3.57 * 10^-4^

*** = p < 3.57 * 10^-5^

**** = p < 3.57 * 10^-6^

*Table S2. Significant differences in the potassium concentration between the various sources of saliva by paired Wilcoxon signed rank test with Bonferroni correction.*

| **Potassium** | Palate | Anterior tongue | Posterior tongue | Upper cheek | Middle cheek | Lower cheek | Floor of the mouth |
| --- | --- | --- | --- | --- | --- | --- | --- |
| UWS | *** | **** | **** | **** | **** | **** | * |
| Palate | - | *** | **** | **** | **** | **** | * |
| Anterior tongue | - | - | NS | ** | *** | NS | **** |
| Posterior tongue | - | - | - | ** | NS | NS | **** |
| Upper cheek | - | - | - | - | NS | *** | **** |
| Middle cheek | - | - | - | - | - | NS | **** |
| Lower cheek | - | - | - | - | - | - | **** |

NS = Not Significant

UWS = Unstimulated Whole Saliva

* = p < 1.79*10^-3^

** = p < 3.57 * 10^-4^

*** = p < 3.57 * 10^-5^

**** = p < 3.57 * 10^-6^

*Table S3. Significant differences in the calcium concentration between the various sources of saliva by paired Wilcoxon signed rank test with Bonferroni correction.*

| **Calcium** | Palate | Anterior tongue | Posterior tongue | Upper cheek | Middle cheek | Lower cheek | Floor of the mouth |
| --- | --- | --- | --- | --- | --- | --- | --- |
| UWS | NS | NS | NS | ** | *** | ** | NS |
| Palate | - | NS | NS | ** | ** | ** | NS |
| Anterior tongue | - | - | NS | NS | ** | * | NS |
| Posterior tongue | - | - | - | NS | * | NS | NS |
| Upper cheek | - | - | - | - | NS | NS | * |
| Middle cheek | - | - | - | - | - | NS | ** |
| Lower cheek | - | - | - | - | - | - | * |

NS = Not Significant

UWS = Unstimulated Whole Saliva

* = p < 1.79*10^-3^

** = p < 3.57 * 10^-4^

*** = p < 3.57 * 10^-5^

*Table S4. Significant differences in the ammonium concentration between the various sources of saliva by paired Wilcoxon signed rank test with Bonferroni correction.*

| **Ammonium** | Palate | Anterior tongue | Posterior tongue | Upper cheek | Middle cheek | Lower cheek | Floor of the mouth |
| --- | --- | --- | --- | --- | --- | --- | --- |
| UWS | ** | **** | **** | NS | NS | NS | *** |
| Palate | - | **** | **** | * | NS | * | ** |
| Anterior tongue | - | - | * | **** | **** | **** | **** |
| Posterior tongue | - | - | - | **** | **** | **** | **** |
| Upper cheek | - | - | - | - | NS | NS | NS |
| Middle cheek | - | - | - | - | - | NS | ** |
| Lower cheek | - | - | - | - | - | - | NS |

NS = Not Significant

UWS = Unstimulated Whole Saliva

* = p < 1.79*10^-3^

** = p < 3.57 * 10^-4^

*** = p < 3.57 * 10^-5^

**** = p < 3.57 * 10^-6^

*Table S5. Significant differences in the chloride concentration between the various sources of saliva by paired Wilcoxon signed rank test with Bonferroni correction.*

| **Chloride** | Palate | Anterior tongue | Posterior tongue | Upper cheek | Middle cheek | Lower cheek | Floor of the mouth |
| --- | --- | --- | --- | --- | --- | --- | --- |
| UWS | **** | **** | **** | **** | **** | **** | NS |
| Palate | - | **** | * | NS | * | * | **** |
| Anterior tongue | - | - | **** | NS | NS | NS | * |
| Posterior tongue | - | - | - | NS | NS | NS | *** |
| Upper cheek | - | - | - | - | NS | NS | **** |
| Middle cheek | - | - | - | - | - | NS | *** |
| Lower cheek | - | - | - | - | - | - | **** |

NS = Not Significant

UWS = Unstimulated Whole Saliva

* = p < 1.79*10^-3^

** = p < 3.57 * 10^-4^

*** = p < 3.57 * 10^-5^

**** = p < 3.57 * 10^-6^

*Table S6. Significant differences in the phosphate concentration between the various sources of saliva by paired Wilcoxon signed rank test with Bonferroni correction.*

| **Phosphate** | Palate | Anterior tongue | Posterior tongue | Upper cheek | Middle cheek | Lower cheek | Floor of the mouth |
| --- | --- | --- | --- | --- | --- | --- | --- |
| UWS | NS | NS | * | **** | **** | *** | NS |
| Palate | - | ** | *** | **** | **** | *** | NS |
| Anterior tongue | - | - | NS | **** | **** | **** | NS |
| Posterior tongue | - | - | - | ** | *** | NS | NS |
| Upper cheek | - | - | - | - | NS | NS | **** |
| Middle cheek | - | - | - | - | - | **** | **** |
| Lower cheek | - | - | - | - | - | - | **** |

NS = Not Significant

UWS = Unstimulated Whole Saliva

* = p < 1.79*10^-3^

** = p < 3.57 * 10^-4^

*** = p < 3.57 * 10^-5^

**** = p < 3.57 * 10^-6^

*Table S7. Significant differences in the nitrate concentration between the various sources of saliva by paired Wilcoxon signed rank test with Bonferroni correction.*

| **Nitrate** | Palate | Anterior tongue | Posterior tongue | Upper cheek | Middle cheek | Lower cheek | Floor of the mouth |
| --- | --- | --- | --- | --- | --- | --- | --- |
| UWS | ** | *** | *** | NS | NS | NS | MS |
| Palate | - | NS | NS | * | NS | NS | NS |
| Anterior tongue | - | - | NS | * | NS | NS | NS |
| Posterior tongue | - | - | - | * | NS | NS | NS |
| Upper cheek | - | - | - | - | NS | NS | NS |
| Middle cheek | - | - | - | - | - | NS | NS |
| Lower cheek | - | - | - | - | - | - | NS |

NS = Not Significant

UWS = Unstimulated Whole Saliva

* = p < 1.79*10^-3^

** = p < 3.57 * 10^-4^

*** = p < 3.57 * 10^-5^

*Table S8. Table depicting the measured median and interquartile range (IQR) of the concentrations of magnesium, nitrite, sulphate and fluoride in unstimulated whole saliva.*

| **Ions** | **Median concentration (IQR) (mM)** |
| --- | --- |
| Magnesium (N = 15) | 0.15 (0.12 – 0.22) |
| Nitrite (N = 3) | 0.33 (0.16 – 1.39) |
| Sulphate (N = 26) | 0.11 (0.08 – 0.16) |
| Fluoride (N = 10) | 0.31 (0.18 – 0.35) |

**
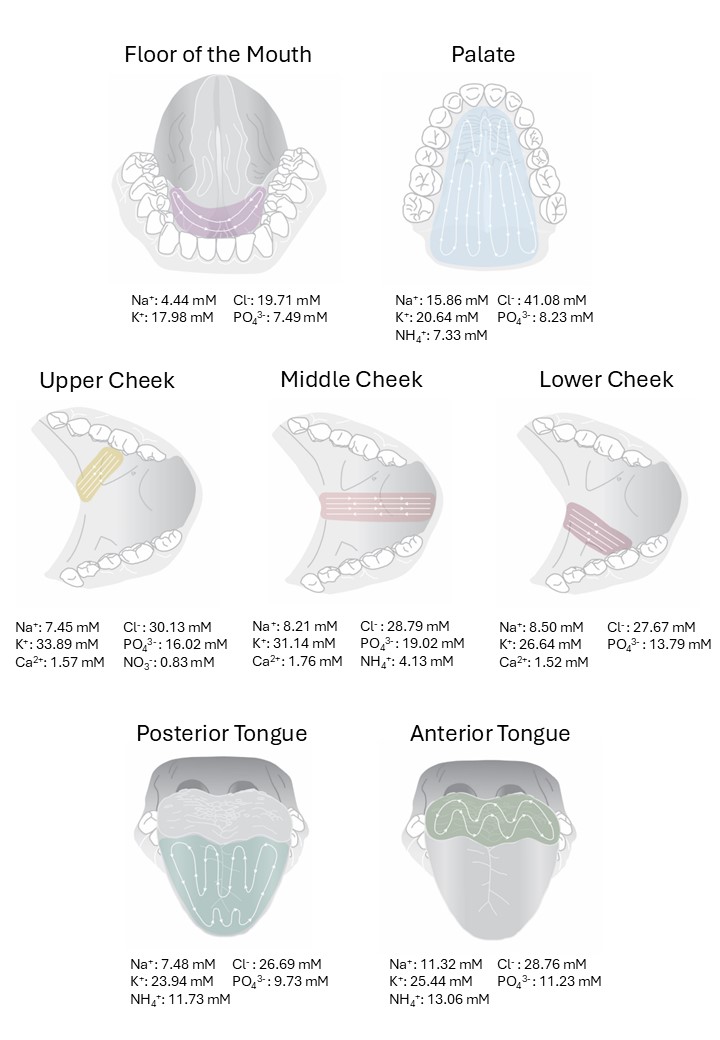
**

*Figure S2*. *The median concentrations of sodium (Na^+^), potassium (K^+^), calcium (Ca^2+^), ammonium (NH_4_^+^), chloride (Cl^-^), phosphate (PO_4_^3-^) and nitrate (NO_3_^-^) at the various intra-oral locations.*

**
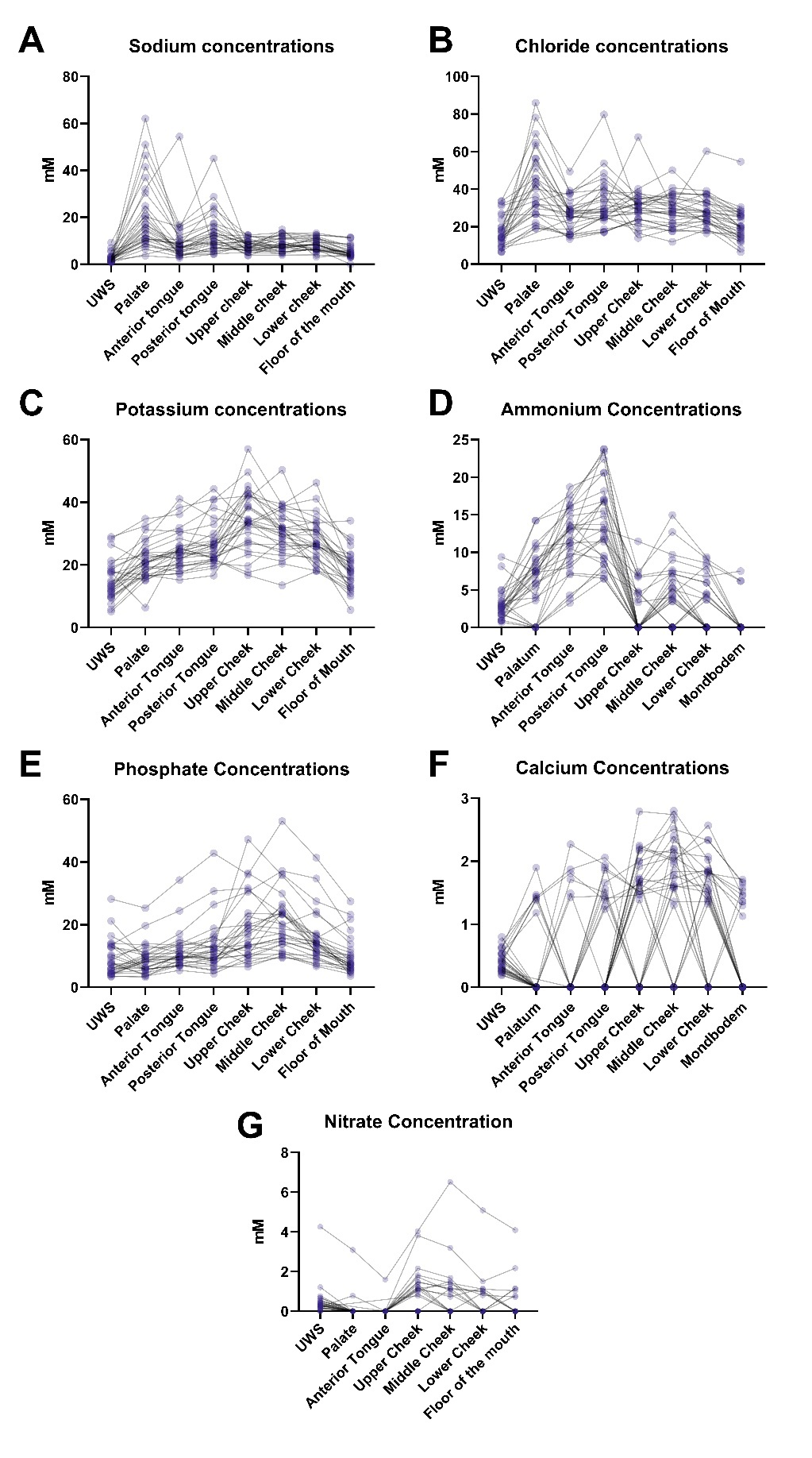
**

*Figure S3. The levels of a.) sodium, b.) chloride, c.) potassium, d.) ammonium e.) phosphate, f.) calcium and g.) nitrate depicted and connected per individual to show the overall trends in concentration change. Any capillary electrophoresis data below the detection limit, were substituted by an arbitrary value of 0.00 mM.*

*Table S9. An overview of the median ion concentration levels in UWS and at the various intra-oral locations.*

|  | **UWS** | **Palate** | **Anterior Tongue** | **Posterior Tongue** | **Upper Cheek** | **Middle Cheek** | **Lower Cheek** | **Floor of the Mouth** |
| --- | --- | --- | --- | --- | --- | --- | --- | --- |
| Sodium | 1.94 | 15.86 | 7.48 | 11.32 | 7.45 | 8.21 | 8.50 | 4.44 |
| Potassium | 13.31 | 20.64 | 23.94 | 25.44 | 33.89 | 31.14 | 26.64 | 17.98 |
| Chloride | 14.49 | 41.08 | 26.69 | 28.76 | 30.13 | 28.79 | 27.67 | 19.71 |
| Phosphate | 7.48 | 8.23 | 9.73 | 11.23 | 16.02 | 19.20 | 13.79 | 7.59 |
| Calcium | 0.39 |  |  |  |  |  |  |  |
| Ammonium | 2.65 | 7.33 | 11.73 | 13.06 | 13.06 |  |  |  |
| Nitrate | 0.30 |  |  |  |  |  |  |  |

Empty fields = The majority of the ion measurements at the specific intra-oral location were below the detection limit.
